# Fabrication of 3D Micro-Blades for the Cutting of Biological Structures in a Microfluidic Guillotine

**DOI:** 10.3390/mi12091005

**Published:** 2021-08-24

**Authors:** Saisneha Koppaka, Kevin S. Zhang, Myra Kurosu Jalil, Lucas R. Blauch, Sindy K. Y. Tang

**Affiliations:** Department of Mechanical Engineering, Stanford University, Stanford, CA 94305, USA; skoppaka@stanford.edu (S.K.); kszhang@stanford.edu (K.S.Z.); mkurosu@stanford.edu (M.K.J.); Luke.blauch@gmail.com (L.R.B.)

**Keywords:** 3D printing, microfabrication, microfluidic guillotine, single cell, wound healing

## Abstract

Micro-blade design is an important factor in the cutting of single cells and other biological structures. This paper describes the fabrication process of three-dimensional (3D) micro-blades for the cutting of single cells in a microfluidic “guillotine” intended for fundamental wound repair and regeneration studies. Our microfluidic guillotine consists of a fixed 3D micro-blade centered in a microchannel to bisect cells flowing through. We show that the Nanoscribe two-photon polymerization direct laser writing system is capable of fabricating complex 3D micro-blade geometries. However, structures made of the Nanoscribe IP-S resin have low adhesion to silicon, and they tend to peel off from the substrate after at most two times of replica molding in poly(dimethylsiloxane) (PDMS). Our work demonstrates that the use of a secondary mold replicates Nanoscribe-printed features faithfully for at least 10 iterations. Finally, we show that complex micro-blade features can generate different degrees of cell wounding and cell survival rates compared with simple blades possessing a vertical cutting edge fabricated with conventional 2.5D photolithography. Our work lays the foundation for future applications in single cell analyses, wound repair and regeneration studies, as well as investigations of the physics of cutting and the interaction between the micro-blade and biological structures.

## 1. Introduction

Wound repair and regeneration are essential biological processes for maintaining homeostasis and survival. Traditionally, wound repair studies have relied on manual surgery [1,2] or laser ablation [3,4,5,6] for generating wounds, but both methods are slow and incompatible with high-throughput analyses (e.g., RNA sequencing), which can require hundreds of samples prepared in a synchronized stage of the repair process. Previously, we have described a microfluidic “guillotine” to automate the cutting of cells [7,8] and organoids [9] in a continuous-flow manner. Instead of moving a micro-blade against an immobilized cell or organoid (referred to as “cellular structure” herein), we flow the cellular structures into a fixed micro-blade, consisting of a triangular wedge, inside a microfluidic channel. This design allows for simple alignment of the cellular structures to the blade, and it facilitates the cutting of a continuous stream of cellular structures in a flow-through manner as the cut structures can be flushed out of the channel easily. Since the cellular structure we used was soft, we found that the blade made of poly(dimethylsiloxane) (PDMS) was sufficient to bisect the cells. The blade, fabricated using conventional 2.5D photolithography and soft lithography, had a simple vertical cutting edge. Due to the limited resolution of the lithography method, the blade was relatively blunt with a radius of curvature of ~10 μm at the tip of the blade.

It is known that the shape of the blade is important in the cutting process and that it affects the extent of wounding and ultimately the viability of the cut cellular structures [10,11,12]. However, conventional 2.5D photolithography does not allow for the fabrication of complex blade shapes. In this paper, we describe the fabrication of a microfluidic guillotine with 3D micro-blades using the Nanoscribe high-resolution two-photon polymerization direct laser writing lithography (TPP-DLW) system. TPP-DLW additive manufacturing spans a wide range of fields and applications [13,14,15,16]. With the Nanoscribe system, we can fabricate complex blade features that have the potential to cut biological material more effectively than simple blades with a vertical cutting edge. We use *Stentor coeruleus* as our model cellular structure for characterizing our fabricated 3D micro-blades. *Stentor coeruleus* is a single-celled ciliated protozoan known to be capable of recovering robustly from drastic wounds and regenerating from cell fragments as small as 1/27th of the original cell size [7,8,17,18,19]. It was also the cell type we used in our previous microfluidic guillotine studies [7,8]. Since the use of two-photon laser lithography for fabricating microfluidic channels is relatively new, this paper focuses on the fabrication and replication process in PDMS. We compare five blade geometries, some inspired by knives used in cutting food, and include a proof-of-concept demonstration of cutting in *Stentor*. The detailed characterization of the physics of the cutting process and its relation to the blade geometry is the subject of an ongoing, separate study.

## 2. Materials and Methods

### 2.1. Microchannel Design

We designed our channels to bisect *Stentor coeruleus* and to prevent them from swimming upstream in the inlet tubing. Each channel consisted of an inlet, an ~1800 μm long and 200 μm wide straight section leading to the micro-blade, and an outlet. The channel confined the cells and aligned them with the micro-blade, which was positioned at the center of the channel. The channel height was 98 μm. In this study, only the micro-blade geometries were varied.

### 2.2. Surface Treatment of Substrates for Adhesion Test

To establish the Nanoscribe system’s ability to fabricate master molds for soft lithography, we characterized the adhesion of the Nanoscribe photoresist to silicon substrates with various surface treatments. We tested the adhesion of photoresist by counting the number of times poly(dimethylsiloxane) (PDMS) (Sylgard 184, Dow Corning, Midland, MI, USA) could be poured onto, cured, and manually peeled off the substrate without the photoresist detaching from the substrate.

Five treatment conditions for silicon wafer substrates were tested. The silicon wafer substrates were boron-doped <100> silicon wafers of thickness 550 ± 25 μm and resistivity 0.1–0.9 Ω-cm. The treatment conditions were (1) an untreated control, (2) oxygen plasma exposure (Matrix Plasma Resist Strip, Matrix Integrated Systems Inc., Richmond, CA, USA) for at least three minutes at 185 °C with RF power at 450 W, a pressure of 3.750 Torr and 40% O_2_, (3) hexamethyldisilazane (HMDS) vapor priming of the silicon wafer after vacuum dehydration of the wafer at 150 °C (YES Oven, Yield Engineering Systems, Fremont, CA, USA), (4) submerging in a mixture of 150 µL of 3-(trimethoxysilyl)propyl methacrylate (M6514, Sigma-Aldrich, St. Louis, MO, USA) and 30 mL of ethanol for 30 min, and (5) plasma exposure according to treatment condition 2, followed by submerging in a mixture 150 µL of 3-(trimethoxysilyl)propyl methacrylate and 30 mL of ethanol for 2 h [20,21].

For all wafer treatment conditions, after the Nanoscribe features were fabricated, we silanized the wafer for at least 45 min with trichloro(1H,1H,2H,2H-perfluorooctyl) silane. All PDMS replicas were fabricated by mixing the PDMS base and curing agent at a 10:1 ratio, pouring the mixture onto the mold, degassing until no visible air bubbles remained, and cross-linking at 65 °C for at least 5 h.

### 2.3. Scanning Electron Microscopy

Micro-blades were imaged using a scanning electron microscope (Apreo, Thermo Fisher Scientific, Waltham, MA, USA) after sputtering with <10 nm of Au/Pd (60:40) to minimize charging.

### 2.4. Confocal Microscopy

Confocal images of the final PDMS microfluidic devices were obtained using an inverted laser scanning confocal microscope (Zeiss LSM 780). The channel was filled with fluorescein isothiocyanate (FITC)-dextran to provide contrast between the channel and the walls. The devices were imaged using a 63× (NA 1.4) oil-immersion objective at an excitation wavelength of 488 nm and a broad emission filter matching the spectra of FITC-dextran. The intensities of each image slice were normalized and inverted in ImageJ and the three-dimensional volume was reconstructed using the ImageJ 3D Viewer plugin.

### 2.5. Stentor Coeruleus Culture and Cell Preparation

*Stentor coeruleus* were cultured in Pasteurized Spring Water, or PSW (132458, Carolina Biological Supply Company, Burlington, NC, USA), in Pyrex dishes in the dark at room temperature. The average diameter of *Stentor* was 400 μm. Algae was cultured under constant light in Tris-acetate-phosphate (TAP) media. We fed *Stentor* cells 1 mL of concentrated algae per 100 mL of *Stentor* culture every other day. To prepare 1 mL of concentrated algae, we spun down 2 mL of *Chlamydomonas* culture that was healthy (with a very green appearance) using a centrifuge (Sorvall Legend Micro 17, Thermo Fisher Scientific, Waltham, MA, USA) at 2× *g*, removed the TAP, washed with 2 mL of PSW, repeated the centrifugation step, and washed with 1 mL of PSW. *Stentor* cultures were fed two days prior to the wounding experiments. To collect cells for the experiments, healthy adult cells (~400 μm in diameter and dark green in color) were retrieved from the culture by pipetting under a stereoscope and into a glass vial.

### 2.6. Experiments on Cutting Stentor Coeruleus

We collected 10 to 15 *Stentor* cells in approximately 25 to 50 μL of PSW into polyethylene tubing (inner diameter of 760 μm) (PE60, Scientific Commodities Inc., Lake Havasu City, AZ, USA) attached to a 3 mL syringe (Covidien 1180300777, Monoject, Thermo Fisher Scientific, Waltham, MA, USA) filled with PSW. Videos of the cutting process were acquired with a high-speed camera (Phantom v341, Vision Research, Inc., Wayne, NJ, USA) mounted on an inverted microscope to count the cells cut and to observe the morphology of the *Stentor* cells being cut.

### 2.7. Measuring Cell Survival

*Stentor* cell survival after cutting was quantified using Equation (1):

(1) $$Survival=\frac{N_{24\;h}}{N_{0\;h}}$$

where $N_{0\;h}$ is the number of cell fragments counted at the channel outlet at t = 0 h, and $N_{24\;h}$ is the number of live cells 24 h after the cut. $N_{0\;h}$ was counted manually from videos of the cutting process and $N_{24\;h}$ was counted manually by collecting cells from the Petri dish where we stored the cells overnight. Cells were considered alive if they had beating cilia, were swimming, or were attached to the surface of the petri dish in a trumpet-like shape that is typical of healthy *Stentor* [7,8,22].

### 2.8. Sytox Green Assay to Measure the Degree of Wounding

To estimate the degree of wounding, we used an indirect measurement based on the fluorescence intensity of a cell-impermeable dye Sytox Green (S7020, Thermo Fisher Scientific, Waltham, MA, USA). We found that on fixed *Stentor* cells, Sytox Green stained wounded cells. The presence of fluorescence indicated the presence of a wound [8]. While this method cannot give an absolute measurement of wound size, a higher fluorescent intensity of Sytox Green stained cells was previously shown to trend with more severe wounding and cytoskeletal damage, as confirmed by tubulin staining of the cytoskeleton. To perform the assay, cells were cut in the microfluidic guillotine, fixed, and then stained with Sytox Green. We used a fixing solution of 1% formaldehyde (no methanol) (43368, Alfa Aesar, Haverhill, MA, USA) and 0.025% Triton X-100 (X100-100ML, Sigma-Aldrich, St. Louis, MO, USA) in PSW at room temperature. Then, 250 μL of cells in PSW was injected through the cell inlet into the microfluidic guillotine device. The outlet tubing led directly into 1000 μL of fixing solution in a 2 mL round bottomed tube, and the cells were fixed for 10 min at room temperature. The length of the outlet tubing controlled the time delay between wounding and fixation. In our experiments, we used a length of 8 cm, corresponding to ~30 s after wounding.

To stain the wounded cells, we used Sytox Green at a concentration of 2.5 μM in PSW. After fixation, we used a 200 μL pipette tip with the end cut off at the 20 μL line to transfer 50 μL of the cells from the bottom of the fixation tube to a 500 μL solution of Sytox in a 4 mL glass vial with a flat bottom (C4015-21, Thermo Fisher Scientific, Waltham, MA, USA). After 30 min of incubation in Sytox, we washed 50 μL of the stained cells in 500 μL of PSW in a second 4 mL glass vial, and then transferred 50 μL of washed cells onto a No. 1 glass slide using a 200 μL pipette tip with the end cut off at the 20 μL line. Cells were then imaged at 15× magnification via epifluorescence using an EMCCD camera (Andor iXon 897, Oxford Instruments, Abingdon, UK) with 0.05 s exposure time, a mercury lamp set to ND 1, and a FITC excitation/emission filter set. To quantify the fluorescence of cells stained with Sytox, we manually traced the cells in ImageJ and measured the average pixel intensity of each cell.

## 3. Results and Discussion

### 3.1. Fabrication of 3D Micro-Blades Using High Resolution Two-Photon Laser Lithography in the Nanoscribe System

Designs for the micro-blades were developed using SolidWorks (Dassault Systèmes, Vélizy-Villacoublay, France). The overall assembly was converted into the STL file format and imported into the computer-aided manufacturing (CAM) software DeScribe (Nanoscribe GmbH, Eggenstein-Leopoldshafen, Germany). The molds were fabricated using the Nanoscribe Photonic GT system. The silicon substrates were prepared as described in Section 2.2. We used the dip-in laser lithography (DiLL) process with a 25× immersion objective (NA 1.4) and IP-S resist on a 525 µm silicon wafer. The 25× objective has a theoretical resolution of 600 nm in the horizontal plane and 3 μm in the vertical direction, sufficient for the generation of sharp edges on our blades. We chose to use the 25× objective instead of a 63× objective (200 nm resolution in the horizontal plane, and 700 nm in the vertical direction) as the resolution was sufficient for our application, and it writes approximately 20 times faster than the 63× objective. To facilitate adhesion of the mold to the silicon wafer, we included a focal spot offset of 2 μm into the wafer.

The Nanoscribe system utilizes two-photon polymerization using a femtosecond laser (center wavelength at 780 nm) focused through an objective, using mirrors to scan the laser through the entire device design [23]. As a result, the fabrication time scales with the size of the device. Fabrication of microfluidic devices with a significant lateral footprint of several cm^2^ can take several hours to fabricate. To reduce writing time, we ran the Nanoscribe system in shell and scaffold mode. This mode reduced the fabrication time by a factor of 2.5 through exposing only the outer shell of the design and an inner scaffold to support the structure. The shell and scaffold mode did not cure all photoresist inside the structure, but it created a solid outer wall which allowed for normal photoresist development to proceed. To develop the structure, we first washed off the uncured resist in a bath of SU-8 developer for up to 20 min, swirling occasionally and checking under the microscope after 10 min. Subsequently, we rinsed the wafer several times with isopropyl alcohol, drying between rinses with inert N_2_ gas. We repeated the rinses until there was no more uncured resist visible. To fully cure the photoresist trapped inside the shell, we performed a post-cure hard bake at 190 °C for up to 5 min. While UV curing at 405 nm is also possible [24], the UV curing system accessible to us took >17 h, likely due to the low power of the UV source. Therefore, we opted to heat cure our structures to increase fabrication turnaround.

**Figure 1:**
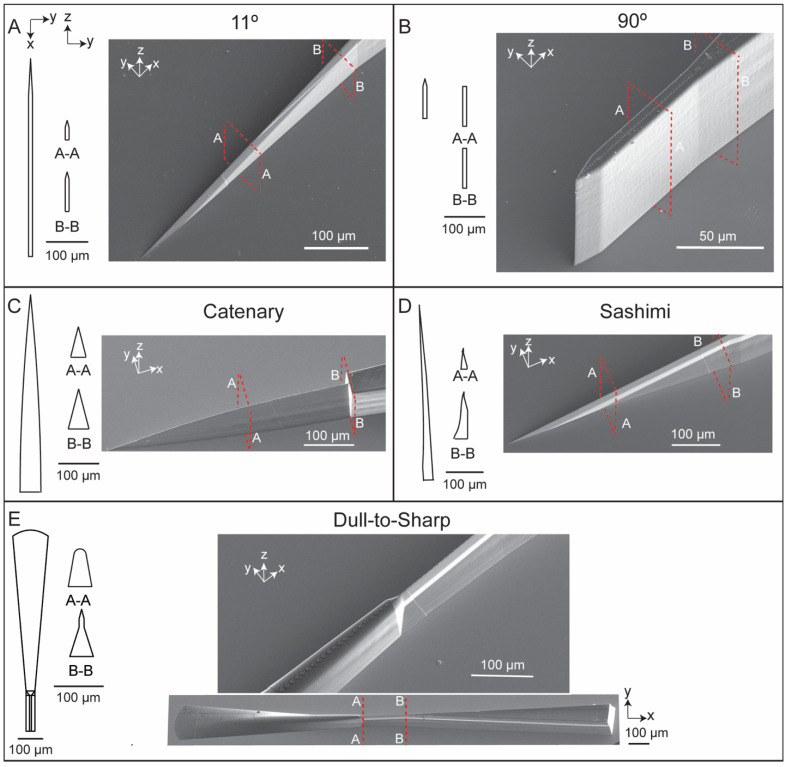
Three-dimensional micro-blades printed using the Nanoscribe Photonic GT system. (**A**) 11°, (**B**) 90°, (**C**) catenary, (**D**) sashimi, and (**E**) dull-to-sharp micro-blade geometries. Cross-section schematic diagrams in the x-y and y-z planes (left) and scanning electron microscope images (right) shown for each micro-blade.

Figure 1 shows the designs and SEM images of five micro-blades fabricated by the Nanoscribe system in IP-S resist. The five geometries are: (i) a blade where the blade edge is straight and has an angle of 11° relative to the horizontal plane (referred to as “11°”), (ii) a blade where the blade edge is straight and has an angle of 90° relative to the horizontal plane (referred to as “90°”), (iii) a catenary blade where the blade edge has a convex shape (referred to as “catenary”), (iv) a blade inspired by a sashimi knife where the sides of the blades are asymmetrical as shown in Figure 1 (referred to as “sashimi”), and (v) a blade where the edge transitions from dull to sharp (referred to as “dull-to-sharp”). These SEM images show that the Nanoscribe system was capable of fabricating complex blade structures that have an aspect ratio of at least 10:1 (height: width).

### 3.2. IP-S Resist Adhesion to Substrates with Different Surface Treatments

Ideally, the Nanoscribe IP-S resist would allow multiple replications in PDMS for making microfluidic channels following typical soft lithography methods that worked well for other photoresists such as SU-8. However, since factors such as solvent permeation and resist adhesion on different substrate materials are not yet fully characterized in the Nanoscribe system, the development and post-cure protocols have remained largely empirical [25]. Therefore, we first characterized the adhesion of IP-S resist to the substrate. To test for adhesion strength qualitatively, we wrote rectangular fin-like features to represent our blade (10 µm wide × 100 µm tall × 500 µm long, see Figure S1) and the inverse of these fins (200 µm wide × 100 µm tall × 1000 µm long, with a slit for the blade structure, see Figure S1) in IP-S using the Nanoscribe system. Table 1 shows the maximum number of PDMS pours on different substrates with different surface treatments before any structures detached as we peeled the cured PDMS off from the substrate. The development and hard bake conditions are also shown in the table. The development and hard bake times were reduced for silicon treated with HMDS and 3-(trimethoxysilyl)propyl methacrylate to avoid undesired burning of the IP-S resist.

The strength of adhesion of IP-S resin were as follows: untreated silicon and silicon treated with plasma enabled two pour/peels, while silicon treated with HMDS or 3-(trimethoxysilyl)propyl methacrylate enabled one pour/peel. After two pour/peels on untreated and plasma treated silicon, or after one pour/peel on silicon treated with 3-(trimethoxysilyl)propyl methacrylate, the fin-like features detached while the inverse structures remained, likely due to the larger surface area of attachment to the substrate in the latter. After one pour/peel on silicon treated with HMDS, both the fin-like features and the inverse structures features detached. In all cases, the maximum number of peels before any structures detached was less than or equal to 2, which was too few for repeated replication in PDMS to make multiple microfluidic devices. While it is possible to further optimize the surface treatment methods to increase the adhesion of IP-S resist, it is beyond the scope of the current study. Instead, we decided to pursue a double replication method to generate the microfluidic channels in PDMS.

### 3.3. Double Replication Process to Generate Microfluidic Channels in PDMS

**Figure 2:**
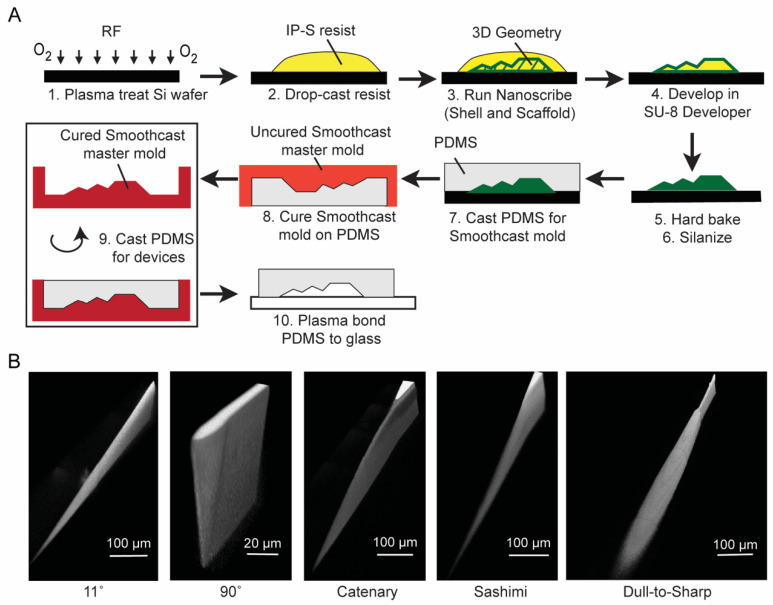
Fabrication and double replication of the micro-blades in a microfluidic channel. (**A**) Process flow to fabricate micro-blades in PDMS. Arbitrary 3D structure depicted. (**B**) Confocal image reconstruction of PDMS replicas of the micro-blades inside a microfluidic channel.

Figure 2A shows our double replication process. Since the surface chemistry of the substrate did not make a significant difference in the adhesion strength, we focused on using a silicon substrate that was treated with oxygen plasma for at least 3 min. IP-S resin was drop-cast onto the wafer, and the micro-blade and microchannel structures were written by the Nanoscribe system as described in Section 3.1. We note that the micro-blades here were the inverse of those in Figure 1 since we are now interested in the replication of the blades in PDMS. After the IP-S features were written and developed, we silanized the surface of the wafer for at least 45 min with trichloro(1H,1H,2H,2H-perfluorooctyl) silane to prevent PDMS from sticking to the wafer and pulling off the IP-S mold. We cured PDMS around this mold. We then made a secondary mold in polyurethane by curing Smooth-Cast 310 (Smooth-On, Inc., Macungie, PA, USA) on top of the PDMS replica [26] in a custom-made pressure chamber with a pressure of ~40 psi for at least 1.5 h at room temperature to remove air bubbles. This secondary polyurethane master could then be used multiple times (at least 10 times in our experiments) to make PDMS replicas, which formed the final devices. Finally, we bonded all PDMS channels to glass slides after exposing both surfaces to oxygen plasma for 2 min.

Figure 2B shows the reconstructed 3D confocal images of PDMS replicas of the micro-blades fabricated using our double replication process. These images were generated by filling the microchannel with a fluorescent dye (see Section 2.4 for details). The micro-blades in PDMS reproduced the shapes of our micro-blades in IP-S (Figure 1) and the original CAD design. We also measured the radius of curvature of the blade tip of the 90° blade in PDMS to be approximately 2 μm (Figure S2). This value agreed approximately with the resolution of the Nanoscribe settings for the 25× objective (3 μm in the z direction and 600 nm in the horizontal direction). Figure S3 shows additional images of the replicated blades.

### 3.4. Cell Cutting Experiments

As a proof of concept of the micro-blade designs, we used the micro-blades to bisect *Stentor* cells. Cells were injected into the device using a syringe pump at a fixed flow rate of 4.5 mL/h or, equivalently, a linear velocity of 6.4 cm/s at the blade. This flow rate corresponded to a regime of cell cutting where large degrees of cell deformation and wounding occurred with reduced cell survival when tested on a vertical blade in our previous publications [7,8].

**Figure 3:**
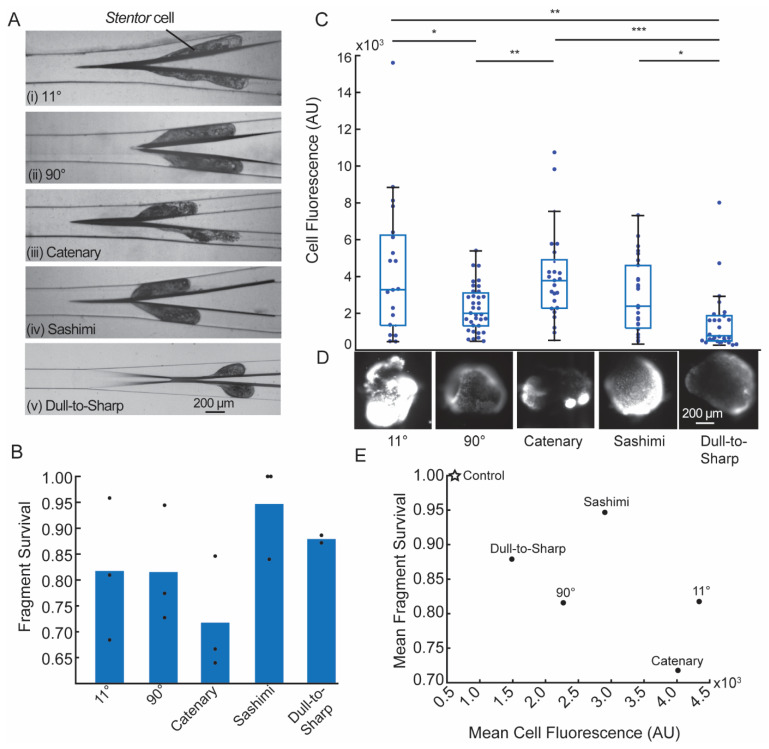
Characterization of the cutting of *Stentor* cells. (**A**) Top-down view (in x-y plane) of the cutting of *Stentor* cell with an average linear velocity of 6.4 cm/s. All images have the same magnification. (**B**) Survival of cell fragments after being bisected by different micro-blade geometries, shown as mean (bar) of *n* ≥ 2 biological replicates (dots). N ≥ 20 cell fragments were counted per replicate. (**C**) Mean Sytox Green fluorescence intensities of cell fragments 30 s after being cut by different micro-blades, shown as box and whisker plots. Individual data points overlaid as a swarmplot. The bar represents the median of the data points. Data for each micro-blade combined from *n* = 2 biological replicates (N ≥ 20 cell fragments per replicate). To assess differences between blade geometries, we performed unpaired two-sample t-tests with Bonferroni correction for multiple comparisons. Significance is denoted using asterisks with * indicating *p* ≤ 0.05, ** indicating *p* ≤ 0.01, and *** indicating *p* ≤ 0.001. (**D**) Representative fluorescence images of *Stentor* cell fragments for each micro-blade geometry. All fluorescence images were scaled equally (100–10,000 AU). Scale bar applies to all images. (**E**) Mean survival as a function of average Sytox Green fluorescence intensity for five micro-blades and the unwounded control condition. Values for the micro-blades are from 3B and 3C. The unwounded control cells (labelled as “Control”) had 100% survival and a mean fluorescence intensity of 610.

Figure 3A shows representative snapshots of the cell being cut by the five blades. Figure 3B shows the mean survival rate of the fragments 24 h after being cut by the blades. Based on these data, the sashimi and the dull-to-sharp blades had the highest mean survival rates (0.95 and 0.88, respectively), while the catenary blade had the lowest survival rate (0.71). The angle of the blade (11° vs. 90°) appeared to make no significant difference in the mean survival rate.

To further examine the effects of the blade geometries, we considered the degree of wounding using an indirect Sytox assay (see details in Section 2.8). Qualitatively, a higher fluorescence intensity corresponds to a larger degree of wounding. Figure 3C shows the distribution of fluorescence intensities of the fragments 30 s after bisection. All fluorescence images were scaled equally (100–10,000 arbitrary pixel units (AU)). Figure 3D shows representative fluorescence images of the cell fragments for each micro-blade geometry. The dull-to-sharp blade had the lowest median degree of wounding, while the catenary blade had the highest median degree of wounding.

The detailed examination of the effect of blade geometries on wounding and survival of the cell is complex, and it is the subject of a separate study. It depends not only on the blade geometry, but also the flow rate and the viscoelasticity of the cell, which is also flow rate-dependent. However, for the experiments performed here, we did notice a negative correlation between the degree of wounding and cell survival (Figure 3E), indicating that cells that were more severely wounded tended to have lower survival rates at 24 h.

## 4. Conclusions

In summary, this work established the successful fabrication of complex micro-blades inside a microfluidic channel in PDMS using a two-photon laser lithography and a double replication process. The adhesion of the IP-S resin to silicon substrates with different surface treatments was low, but this issue was resolved by using a secondary mold made of polyurethane. We showed that the micro-blades were able to cut *Stentor* cells, and that different blade geometries resulted in different degrees of cell wounding and survival rates. It was difficult to predict the effect of the blade geometry due to the heterogeneity of the mechanical properties of the cell and its complex interaction with the blade. Nevertheless, this work established the groundwork for future systematic investigations of the effect of the blade geometry on the cutting process, including the use of particles with well-characterized mechanical properties and varying the applied flow rate.

## Figures and Tables

**Table 1:** Qualitative adhesion test based on the number of times that PDMS can be peeled off before IP-S resist detaches from the substrate.

Substrate	Development Time (Minutes)	Hard Bake Time at 190 °C (Minutes)	Number of Times PDMS Can Be Cured and Peeled off without Detaching Any IP-S Features from the Substrate
**Si**	16	5	2
**Si + Plasma treatment**	16	5	2
**Si + HMDS**	15	2	1
**Si + 3-(trimethoxysilyl)propyl methacrylate (30 min)**	10	0.5	1
**Si + Plasma treatment +** **3-(trimethoxysilyl)propyl methacrylate (2 h)**	10	0.5	1

## Data Availability

The data that support the findings of this study are available from the corresponding author upon reasonable request.

## Supplementary Materials

The following are available online at https://www.mdpi.com/article/10.3390/mi12091005/s1. Figure S1: Schematic diagrams of the rectangular features for IP-S photoresist adhesion tests. Figure S2: Radius of curvature of the tip of the 90° blade. Figure S3: Confocal image reconstruction of PDMS replicas of the micro-blades inside a microfluidic channel showing isometric, top-down, and cross-section views. Table S1: Table of Bonferroni corrected *p*-values of Sytox fluorescence data.
