# Supplementary material for: Fabrication of 3D Micro-Blades for the Cutting of Biological Structures in a Microfluidic Guillotine
Source: Micromachines (Basel). 2021 Aug 24;12(9):1005. doi: 10.3390/mi12091005 (PMC8472695; doi:10.3390/mi12091005)
Supplement: Supplementary file 1 [file micromachines-12-01005-s001.zip › micromachines-1334171-supplementary.pdf]

Supplementary Materials: Fabrication of 3D micro-blades for the cutting of biological structures in a microfluidic guillotine

Saisneha Koppaka, Kevin S. Zhang, Myra Kurosu Jalil, Lucas R. Blauch and Sindy K. Y. Tang

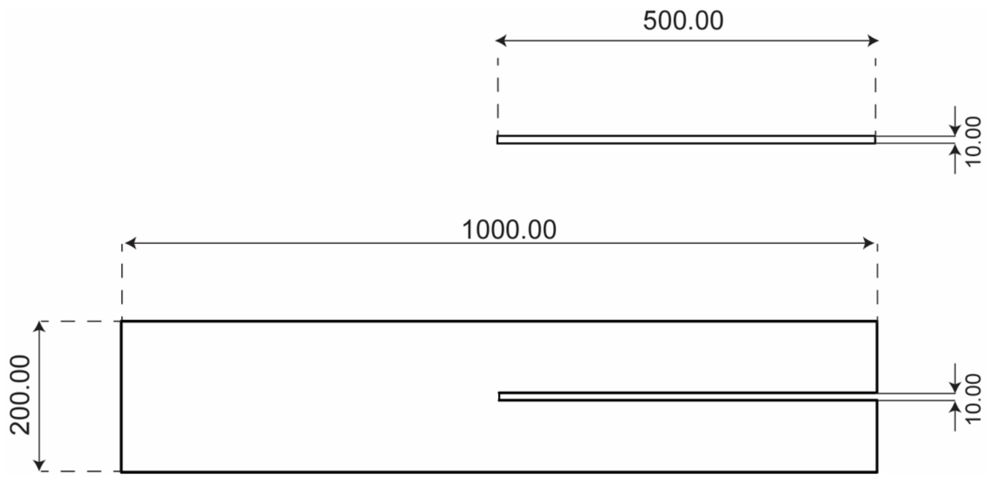

Figure S1. Schematic diagrams of the rectangular features for IP-S photoresist adhesion tests. Top view of rectangular features and the inverse of these features for the adhesion test in Table 1 (main text). Both features are 100 μm tall. All dimensions are in units of microns.

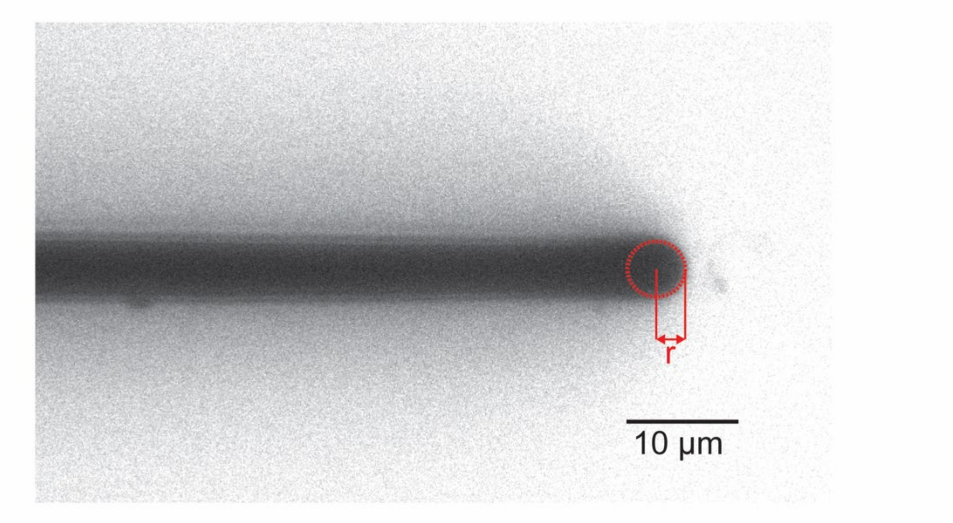

Figure S2. Radius of curvature of the tip of the 90° blade. Confocal microscope image of the 90° blade in PDMS used for measuring blade tip radius. See section 2.4 in the text for details.

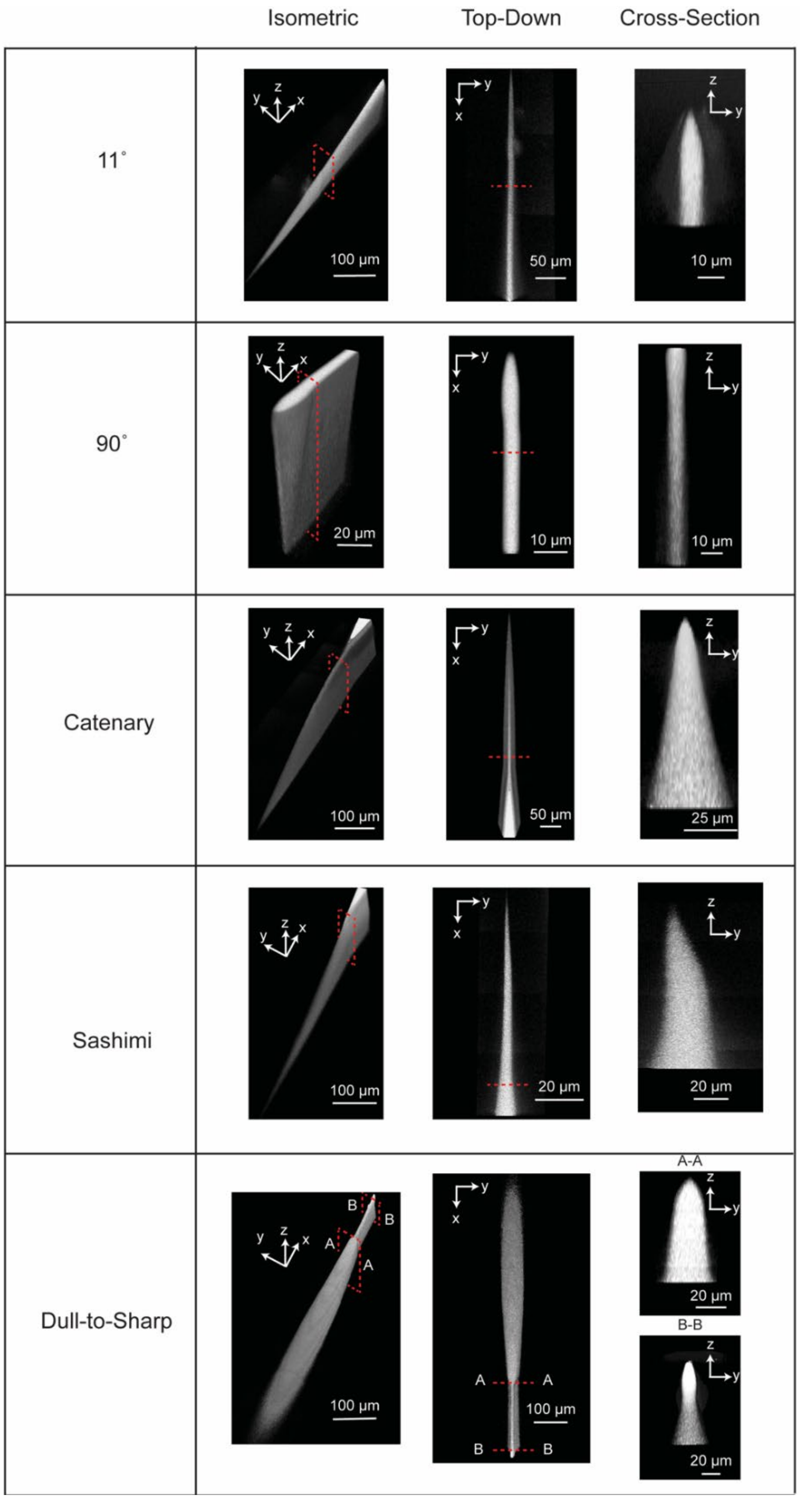

Figure S3. Confocal image reconstruction of PDMS replicas of the micro-blades inside a microfluidic channel showing isometric, top-down, and cross-section views.

Table S1. Table of Bonferroni corrected p-values of Sytox fluorescence data. Significance is denoted with * indicating $p \leq 0.05$, ** indicating $p \leq 0.01$, and *** indicating $p \leq 0.001$.

	**90°**	**11°**	**Catenary**	**Sashimi**	**Dull to Sharp**
**90°**		0.041*	0.007**	1.000	0.340
**11°**			1.000	0.797	0.008**
**Catenary**				0.669	0.0005***
**Sashimi**					0.038*
**Dull to Sharp**					
